# From Broca and Wernicke to the Neuromodulation Era: Insights of Brain Language Networks for Neurorehabilitation

**DOI:** 10.1155/2019/9894571

**Published:** 2019-07-22

**Authors:** Grigorios Nasios, Efthymios Dardiotis, Lambros Messinis

**Affiliations:** ^1^Department of Speech and Language Therapy, University of Ioannina, Ioannina, Greece; ^2^Department of Neurology, University Hospital of Larisa, University of Thessaly, Larisa, Greece; ^3^Neuropsychology Section, Departments of Neurology and Psychiatry, University of Patras Medical School, Patras, Greece

## Abstract

Communication in humans activates almost every part of the brain. Of course, the use of language predominates, but other cognitive functions such as attention, memory, emotion, and executive processes are also involved. However, in order to explain how our brain “understands,” “speaks,” and “writes,” and in order to rehabilitate aphasic disorders, neuroscience has faced the challenge for years to reveal the responsible neural networks. Broca and Wernicke (and Lichtheim and many others), during the 19th century, when brain research was mainly observational and autopsy driven, offered fundamental knowledge about the brain and language, so the Wernicke-Geschwind model appeared and aphasiology during the 20th century was based on it. This model is still useful for a first approach into the classical categorization of aphasic syndromes, but it is outdated, because it does not adequately describe the neural networks relevant for language, and it offers a modular perspective, focusing mainly on cortical structures. During the last three decades, neuroscience conquered new imaging, recording, and manipulation techniques for brain research, and a new model of the functional neuroanatomy of language was developed, the dual stream model, consisting of two interacting networks (“streams”), one ventral, bilaterally organized, for language comprehension, and one dorsal, left hemisphere dominant, for production. This new model also has its limitations but helps us to understand, among others, why patients with different brain lesions can have similar language impairments. Furthermore, interesting aspects arise from studying language functions in aging brains (and also in young, developing brains) and in cognitively impaired patients and neuromodulation effects on reorganization of brain networks subserving language. In this selective review, we discuss methods for coupling new knowledge regarding the functional reorganization of the brain with sophisticated techniques capable of activating the available supportive networks in order to provide improved neurorehabilitation strategies for people suffering from neurogenic communication disorders.

## 1. Introduction

Let us consider two healthy men, without a history of neurological disease, one 20 and one 80 years old, who both participated in a sentence comprehension task and showed the same success at comprehending sentences. The older individual is able (under circumstances) to perform similarly with the younger one, due to the compensatory recruitment of novel, ancillary brain regions [1]. These ancillary regions although available are not necessary in the younger person's brain. This phenomenon, which cannot be observed clinically but can be captured by functional resonance imaging, is “hidden” presbyphasia and in simple words tells us that an aged brain requires more effort to perform similarly to the younger one. The above simple paradigm offers a new perspective for the neuroanatomy of language and can be observed under various points of view: the brain's multifunctionality, its structural and functional connectivity, plasticity, and cognitive reserve hypothesis. It helps us to see the whole brain as an organ of communication, where linguistic and cognitive networks uninterruptedly cooperate. In our recently published editorial, we highlight, based on evidence from healthy and diseased brains, its ability to make communication possible through meaningful symbols, expressions, and comprehension of ideas and concepts [2]. This selective review begins with a brief description of the Broca-Wernicke-Lichtheim-Geschwind classical model, from the era of autopsy-driven research. Then, Hickok and Poeppel's dual stream model of language processing is described, including its limitations (i.e., the role of the cerebellum). It then continues with a discussion of the ability to use language to communicate, which relies not only on the traditionally described core language networks but also on other additional, widely distributed, networks, which can be recruited to support linguistic functions when needed. New insights are offered by studying language in the aging brain and also language in young/developing brains. We also comment on the linkage between language and cognition especially in the elderly, as well as in poststroke aphasia, and the reorganization of language networks. Describing these networks, testing for their availability, and enhancing their recruitment constitute the science of neurorehabilitation. The newly conquered tools for clinical and experimental applications of neuroimaging and neuromodulation, i.e., artificial manipulation of brain activity, assisted neuroscience to establish a new era. By utilizing these modern methods, we have advanced our knowledge and subsequently challenged the classical knowledge about the brain and language. In the final part of this paper, we discuss methods for coupling the obtained new knowledge with sophisticated techniques in order to advance neurorehabilitation. We intentionally highlight numerous aspects of brain and language associations that could give rise to ideas on new therapeutic targets for people living with aphasia.

## 2. The Broca-Wernicke-Lichtheim-Geschwind Classical Model

Broca (1824-1880) first described in 1861, after autopsying the brain of his famous patient “Tan” (Louis Victor Leborgne), the association between motor aphasia and a lesion in the middle part of the patient's left frontal lobe, the cortical speech center, an area later named after him, as “Broca's area” [3, 4]. Shortly after Broca published his findings, Wernicke (1848-1905) noticed that not all language deficits were the result of damage to Broca's area. He observed in 1873 that damage to the left posterior superior temporal gyrus, now referred to as Wernicke's area, resulted in deficits in language comprehension, an aphasia later known as Wernicke's aphasia (receptive aphasia) [5]. Ludwig Lichtheim (1845-1928) described thoroughly the elements of conduction aphasia and developed an explanation of language processing in the brain. Furthermore, he developed an early model about the neuroanatomy of language, the so-called Wernicke-Lichtheim model. Later, during the 20^th^ century, Norman Geschwind (1926-1984), a pioneering American behavioral neurologist, revived the Wernicke-Lichtheim model for the neuroanatomy of language so that the model is more widely known as the “Wernicke-Geschwind” model. According to this model, the sounds of the words are transferred through the auditory pathways to the primary auditory cortex and then to Wernicke's area, where the meaning of the words is extracted. In order for a person to produce speech, the meanings of words are sent from Wernicke's area via the arcuate fasciculus to Broca's area, where morphemes are formed and then passed on to the motor cortex. On the other hand, information from the written word is transferred through the primary visual cortex to the angular gyrus and then to Wernicke's area. To do justice, we should refer to the model that predominated for more than a century as the standard neurological model of language, the Broca-Wernicke-Lichtheim-Geschwind model.

In this historical model, Wernicke's and Broca's areas are connected to each other by the arcuate fasciculus. Where exactly Broca's and Wernicke's areas are located in the brain is also a matter of ambiguity, especially for the latter. Broca's area corresponds to the triangular and opercular inferior frontal gyrus (IFG) of the left hemisphere for the majority of humans. Dronkers and colleagues, reinspecting with high-resolution magnetic resonance imaging the preserved brains of Broca's two historic patients, found that both patients' lesions extended significantly not only to the surface lesions, originally observed by Broca, but also into medial regions of the brain [6]. Furthermore, Fedorenko and colleagues presented evidence from single-subject fMRI studies suggesting that Broca's area contains two sets of subregions, one specific for language, surrounded by another, nonlanguage-specific, engaged in a wide range of cognitive tasks [7]. One could argue this in favor of the brain's multifunctionality.

As for Wernicke's area, the ambiguity is even higher, with Mesulam and colleagues arguing that there is no single area in our brain dedicated to speech comprehension [8]. Studying a large group of primary progressive aphasia patients, they found a heterogeneous set of cortical atrophy sites associated with severe comprehension impairments for sentence production, including Wernicke's area, Broca's area, and the dorsal premotor cortex, while severe comprehension impairments for single words were associated with atrophy sites in the left temporal pole and adjacent anterior temporal cortex.

The simplified modular approach of the classical model is perhaps still useful for understanding the classical categorization of aphasic syndromes, in which frontal lesions cause motor aphasias, temporal and temporal-parietal lesions cause sensory aphasias, lesions affecting the arcuate fasciculus cause conduction aphasia, and deeper cortical lesions cause disconnection syndromes. This model which dominates in many classical (and not necessarily old) textbooks, and guided research until the beginning of this century, is now outdated. It proved that “it is linguistically and anatomically underspecified” [9] and could not scientifically support the full range of aphasic syndromes. Trembley and Dick [10] reviewed the serious gaps of the classical model and commented that one of the most important problems was “the lack of circuit information regarding the neural connections of the brain areas involved.” One other major concern is that this model focuses on cortical structures, excluding subcortical regions and relevant connections, based on an outdated brain anatomy. During the last three decades, studies based mainly on functional neuroimaging provided proof that during comprehension, both temporal lobes were involved, and during speech production, a wide range of frontal and parietal regions, usually in the left hemisphere, are activated. Furthermore, many subcortical regions and tracts are involved. Interestingly, Behroozmand et al. provide novel evidence that the subthalamic nucleus is involved in vocal motor function as demonstrated after deep brain stimulation treatment of patients with PD [11], forming together the core language networks.

## 3. Hickok and Poeppel's Dual Stream Model and the Language Processing Networks

A fundamental shift away from the older models to modern network-based models was made possible through new knowledge from observations on both human and nonhuman primates [12]. Modern network-based models are composed of parallel and interconnected streams, involving both cortical and subcortical areas. Hickok and Poeppel [13, 14] proposed the “dual stream” model, emphasizing speech processing in “dorsal” and “ventral” pathways (streams): the ventral stream is largely bilaterally organized from the temporal pole to the basal occipitotemporal cortex, processing speech signals for comprehension, while the dorsal stream is strongly left hemisphere dominant, from the posterior superior temporal to the inferior frontal cortices [15]. The function of the dorsal stream is mainly restricted to the “sensory-motor mapping of sound to articulation” [16].

According to the dual stream model, the dorsal pathway involves left hemispheric structures in the posterior frontal lobe, the posterior dorsal temporal lobe, and the parietal operculum, including long white matter (WM) tracts connecting the frontal to the temporal and parietal lobes, namely, the articulate fasciculus (AF), and the indirect anterior and indirect posterior components of the superior longitudinal fascicle (SLF). More specifically, the core anterior (frontal) hubs of the dorsal pathway include the inferior frontal gyrus (opercular and triangular part), the ventral portions of the precentral gyrus, and the anterior portions of the insula (forming together the left frontal operculum (L-FO)). Posteriorly, the main hubs are the posterior sector of the insula, the ventral portions of the supramarginal gyrus, and Sylvian parietal temporal region (Spt), forming, together with the upper parts of the posterior superior temporal gyrus and sulcus, the left temporoparietal junction (L-TPJ).

Area Spt is located in the posterior part of the left planum temporale (PT) region, where speech perception and production systems converge. Despite its proximity to classical Wernicke's area, its posterior part is mainly involved in speech production, acting as a “computational hub” and a sensorimotor interface between the two streams.

On the other hand, ventral pathways are bilaterally distributed into both hemispheres, and the major hubs include the superior temporal gyrus (STG), superior temporal sulcus (STS), middle and inferior temporal gyri (MTG/ITG), and the anterior temporal lobe (ATL). The ventral stream connects the frontal cortices to the occipital, parietal, and temporal lobes, via long white matter (WM) tracts, including the external capsule (EC), the inferior fronto-occipital fascicle (IFOF), the inferior longitudinal fascicle (ILF), and the uncinate fascicle (UF). The main hubs and fascicles constituting the dual stream model are summarized in Table 1.

Although the dual stream model describes the anatomical foundations of normal, and not disordered, speech and language processing, studies from stroke patients with aphasia offered evidence supporting it. Kümmerer et al. assessed, in a large sample of 100 aphasic stroke patients, how well acute impairments of repetition and comprehension correlate with lesions of either the dorsal or ventral stream [17]. They concluded that task performance on auditory comprehension measures requires an interaction between temporal and prefrontal brain regions via the ventral extreme capsule pathway. Fridriksson et al. also examined the effect of both cortical damage and disconnection on aphasic impairment in stroke patients, in the context of this model [18]. They found that “measures of motor speech impairment are more strongly associated with damage to the dorsal stream, whereas measures of impaired speech comprehension with ventral stream involvement.” Interestingly and importantly, they showed that language functions such as naming, speech repetition, and grammatical processing rely on a broader network and on interactions between the two streams. Their results offer evidence from brain-damaged patients supporting the dual model, by linking motor speech impairment mostly with damage to the dorsal stream and impaired speech comprehension with ventral stream involvement. Furthermore, they showed that elements such as naming, speech repetition, and grammatical processing rely mainly on connections and interactions between the two streams. This explains why patients with seemingly disparate lesion locations often experience similar impairments on specific speech or language tasks: they may have dissimilar cortical damage, but this damage affects the same broad cortical network that supports these tasks. Overall, these findings help us to move from a nodular to a network perspective. Of course, there are key regions, in other words, crucial central nodes or hubs, many of them described by the 19th century aphasiologists, but the way they connect and interact with other nodes and tracts provides our brain with its unique capacity for language processing and communication. In addition to these hubs, language networks also include important “bottleneck” areas, referred to below.

A novel synthesis of old and new knowledge about the architecture of the language processing network, in alignment with the dual stream model, is provided by the work of Mirman et al. [19]. In this inspired study, the researchers combined high-quality structural neuroimaging analysis techniques and extensive behavioral assessment of 99 patients with persistent acquired language deficits, in order to answer questions about the functional and neural organization of language processing in patients with acquired language deficits. Two major divisions within the language system can be identified: one serving “meaning versus form” and one for “recognition versus production.” The peri-Sylvian regions involved in phonological processing were either supra-Sylvian (for speech production—in agreement with the dorsal stream) or infra-Sylvian (for speech recognition—in agreement with the ventral stream). On the other hand, semantic production and recognition deficits were linked with damage in extra-Sylvian regions. More specifically, semantic production deficits, reflected in semantic errors, were linked to the left anterior temporal lobe (ATL), while semantic multimodal recognition deficits (were linked) to impaired white matter connectivity of other brain regions with the frontal cortex, respectively. Mirman et al. with their work highlighted the importance of mainly three tracts beyond the arcuate fasciculus: the uncinate fasciculus, the inferior fronto-occipital fasciculus, and the anterior thalamic radiations. All these fiber tracts converge in a “bottleneck” frontal white matter region, where even nonsevere damage can impair semantic processing across multiple tasks and modalities. Outside this bottleneck region, more extensive lesions would be required to produce comparable multimodal semantic deficits. This is why lesions in other regions known to be important for semantic processing, such as the middle and posterior portions of the middle temporal gyrus and the inferior parietal lobule, were not associated in this study with either semantic errors or multimodal semantics. The role of these regions is neither specific to semantically driven word production (that role is played by the left ATL) nor general enough for focal damage to produce multimodal semantic deficits, as is the white matter bottleneck region.

## 4. The Role of the Cerebellum

As we noted previously, the cerebellum does not appear either in the classical or in the modern models describing the neuroanatomy of language. This is not what really happens, however. Cerebellar lesions are reported to cause aphasia [20], and the cerebellum's role in a wide range of nervous system cognitive and affective functions, among them language, has been revealed [21]. A topologic distinction has been established between the “motor” cerebellum, projecting to the cortical motor areas, and the “cognitive/affective” cerebellum, connected with the cortical and limbic association areas [22]. Cerebellar lesions have a remote functional impact on structurally intact cortical regions via crossed cerebello-cerebral diaschisis. Mariën and Borgatti [22] discuss about (strongly lateralized) involvement of the cerebellum in a broad spectrum of nonmotor language functions through a dense network of crossed and reciprocal cerebello-cerebral connections. Recently, the cerebellum was targeted with tDCS (transcranial direct current stimulation) with enhancing effects on verbal fluency [23]. It is time therefore to include the cerebellum in a new, updated, “multiple stream model” of language processing.

## 5. Beyond Networking: Communication and Our Brain

Many neuroscientists, from neuroanatomists to neurolinguists, focus their research on specific small particles of language processing. These efforts together contributed deep inside our brains as communication organs and form our current knowledge on language functions and cognitive-linguistic interactions both in health and disease. An example on this is the findings from one of our recent studies, in which we examined verb-noun dissociations in patients with relapsing remitting multiple sclerosis and reported for the first time in the literature a noun superiority over verbs for picture confrontation naming in these patients [24]. However, in the real world, our brain seems to organize its interactions for communication under scenarios involving multifunctional processing of multimodal input from the environment. In simple words, what we are prepared to listen to is preformed depending on whether we are discussing with friends or attending a scientific lecture. Different groups of words, forming different groups of meanings, connected with different emotional aspects, involve different parts of our brain, far beyond the strictly defined language networks. Huth and colleagues shifted our thinking to this real world using a novel generative model to create a detailed semantic atlas [25]. They wanted to answer questions about the extension and the selectivity of brain regions involved in the representation of the meaning of language. With voxel-wise modeling of functional MRI (fMRI) data, they found that words related to the same semantic domain were highly correlated, while nonrelated words were not. Doing this for several hundreds of words, using a large corpus of English text of narrative stories, subjects were listening to for hours, while in the scanner, they showed that most areas within the semantic system represent information about specific semantic domains. In this way, they were able to form an atlas showing which domains are represented in each area. Additionally, they proved that the semantic system was highly consistent across the participating individuals, and they explained this consistency due to their common life experiences. One other major finding of this genius study was that the distribution of semantically selective areas within the semantic system was relatively symmetrical across the two cerebral hemispheres. This latter finding is inconsistent with human lesion studies that support the idea that semantic representation is lateralized to the left hemisphere but, however, in alignment with the bilateral distribution of the ventral stream of the dual stream model. The bilateral distribution of semantic representation, in other words the extension of the ventral stream into both hemispheres, is strongly supported by numerous studies, since neuroimaging technology allowed us to study language in healthy individuals, as Price excellently reviewed [26, 27].

When we communicate, words predominate in our message exchange network, but we never only exchange words. Different brain areas scan prosody, emotions, body language, and various environmental stimuli. There are no simple rules about the hierarchy of what is more important for our brain in order to “understand” a message. Evidence to help us unravel this hierarchy can be obtained by studying brain-lesioned patients, and this could guide neurorehabilitation. We admire the way Sacks, before the functional imaging and brain networking era, described in the 9th chapter (“The President's Speech”) of his book *The Man Who Mistook His Wife for a Hat* that only the people who were unable to understand words were able to really understand the speaker [28]. We (GN) are now preparing a case report of a 67-year-old man who after an excellent recovery from his aphasia due to a left hemispheric (mainly temporal) stroke ten years ago reappeared a few months ago with severe aphasia, after a right temporal stroke. Could this be interpreted as a case of “aphasia due to right hemispheric lesion” in a patient with a proven “left dominant brain”? Are we justified to answer “no”? Turkeltaub et al. reported a similar very interesting case of a 72-year-old aphasic woman who experienced beneficial effects in naming after being treated with an inhibitory TMS protocol targeting her right pars triangularis, until a new, right hemispheric stroke caused worsening of her aphasia [29]. These cases bring in mind the historical Barlow 1877 case [30], while Hamilton and colleagues review similar other reported cases trying to illuminate the right hemisphere's role in poststroke aphasia recovery [31]. How can these paradoxical observations be explained? Why, even though we know that right hemisphere homologue areas are active during language tasks, do lesions in these areas not typically (but exceptionally) cause language impairment? It may not be the inhibited or lesioned right hemispheric areas per se that matter more but their role within the language networks. When the core left hemispheric language areas are intact, many right homologue ones remain “silent,” but in left hemispheric-lesioned brains, their role appears to be protagonistic. If also these right hemispheric areas are destroyed (as in Turkeltaub et al.'s patient—and an ischemic lesion could not be equivalent to a TMS-induced inhibition), aphasia worsens.

As Cahana-Amitay and Albert point out in their excellent book *Redefining Recovery from Aphasia*, a multifunctional neural model of language should be formed, including brain language mappings that reflect the functional diversity of the neural networks subserving language and the role of nonlinguistic skills [32]. Of course, by establishing the brain areas which are responsible for various task conditions, we do not reach the end of the road. Neuroscience's goal still remains to explain how the brain does it [33].

## 6. Language and the Aging Brain

Investigating possible effects of aging on the brain's structural and functional characteristics is not only important for better understanding neuroplasticity changes of language networks but also due to the fact that the majority of patients with aphasia (due to stroke or neurodegenerative diseases) are elderly. However, current knowledge on brain language maps is typically based on neural data collected from young healthy adults (usually college-aged students), whose functional neuroanatomy is likely distinct from that of older adults in important ways [32].

It has been argued that even though normal aging impairs specific aspects of language production, most core language processes are robust to brain aging [34]. However, as it has recently been reported, young people outperform their older counterparts during semantic tasks [35]. Older adults display less activation than young people in some elements of the typical left hemisphere semantic network but greater activation in right frontal and parietal regions, principally when they perform more poorly than the younger participants. Thus, semantic processing in later life is associated with a shift from semantic-specific to other neural resources.

Agarwal et al. compared two groups of younger and older adults with equivalent cognitive performance, during a word retrieval task, with a multifaceted approach, integrating structural and functional imaging data, on interactions between hemispheres or reorganization of dominant hemisphere networks, resulting in preservation of function [36]. The authors demonstrated that left hemispheric language areas showed higher functional connectivity in older adults with intact behavioral performance and, thus, may have a role in preserving function. Successful language ability among older adults depends on sparing of cognitive abilities, where “the combined contribution of preserved cognitive functions reflects a compensatory mechanism recruited to support a given compromised linguistic function” [32]. Whether this reorganization and overactivation of various, ancillary networks always represent a “successful” compensation, or not, is of course a question that remains unanswered [37]. One could speculate that cognitive decline, especially as far as executive functions are concerned, could be the most relevant explanation of language decline in the elderly and perhaps vice versa. Language keeps the brain active in many aspects, for example, bilingual older adults typically have better performance on tasks of executive control than do monolingual peers. Grady et al. studied brain network activity in monolingual and bilingual older adults and found that bilinguals showed stronger correlations than monolinguals between intrinsic connectivity in the frontoparietal control network and task-related increases of activity in prefrontal and parietal regions [38]. The authors conclude that bilingual differences in network connectivity suggest that language experience begun in childhood and continued throughout adulthood which influences brain networks in ways that may provide benefits in later life. These benefits protect or enhance the brain's executive control systems and may delay the onset of Alzheimer's disease symptoms [39]. This provides further support for multifunctional, mutually interacting perspectives, whereby a constant and dynamic interaction exists among neural networks subserving cognitive, affective, and praxic functions on the one hand and linguistic functions on the other. Of course, further studies, not only in the elderly but also through the entire lifespan, are needed to track the changes that occur in the brain mechanisms underlying cognitive and linguistic processes [37].

## 7. A Note for Young/Developing Brains and Reorganization of Language Networks

Research not only on the aging but also on the developing brains offers insights on various aspects on brain reorganization of language functions. One aspect is the evolution and improvement of linguistic proficiency during the first years of life. Lidzba et al. [40] studied language representation and lateralization in 36 children, adolescents, and young adults, using a language comprehension and a language production task during fMRI. They found that the neural basis of language comprehension is established in a bilateral network by late childhood, while for the productive network, an increase with age both in focus and lateralization was seen. Furthermore, in 24% of their sample, language comprehension and language production were lateralized to opposite hemispheres. Another very interesting aspect was the way that congenital left hemispheric brain lesions were compensated for, as language is concerned. This question is of special interest since previous work has shown that children with congenital left hemisphere damage scored far better in language production tasks than left hemisphere-damaged adults [41]. Another interesting point is that perinatal stroke patients exhibit different patterns of reorganization than childhood stroke patients. Childhood stroke patients showed left-side cortical activation, similar to controls, while perinatal stroke patients showed atypical right-side or bilateral language lateralization [42]. What Lidzba et al. found in their study [40] which corresponds to the dual stream model is that language comprehension is represented more bilaterally than language production. Moreover, a hemispheric dissociation with left hemispheric language production but bilateral or right hemispheric language comprehension is not uncommon even in healthy right-handed subjects.

The Tübingen group addressed the question of how congenital left hemispheric brain lesions affecting language were compensated for [43]. They tried to link lesion characteristics and interhemispheric reorganization, depending on the level of reorganized language production, and comprehension. They observed that patients with lesions in areas belonging to the dorsal stream of the language network such as the insular cortex and the temporoparietal junction (TPJ) showed reorganization of both language production and comprehension more frequently. In other words, the integrity of the dorsal stream might be crucial for a normal left-lateralized language development. The finding that damage to the structures of the dorsal network and not the ventral may lead to reorganization of the networks that support language may be explained by the unilateral dominance (usually left side) of the dorsal network as compared to the bilateral organization of the ventral network. As a consequence, a congenital lesion of the dorsal language pathway in the left hemisphere may induce interhemispheric reorganization of the entire language network, including language comprehension [43]. As regards the TPJ area, although structurally it is placed between the dorsal and ventral networks and despite its proximity to the classical Wernicke's area, its posterior part is mainly involved in speech production. For a detailed description of this area, refer to the book by Hickok and Small [44].

## 8. The Role of Neurorehabilitation for Recovery from Aphasia

Aphasias, the acquired language disorders occurring after disruption of language networks, are a common consequence of stroke and other brain disorders and traditionally are treated by speech and language therapy. The classical speech and language therapy approaches are purely linguistic and vary mainly in the intervention methodology, its duration, intensity, and frequency [45]. These approaches however do not do justice to patients recovering from aphasia. First of all, left hemispheric stroke patients with aphasia may also have cognitive deficits, especially on working memory and sustained attention, and there is an established association between them and aphasia severity [46]. Further evidence for the relationship between linguistic and cognitive functions in patients with poststroke aphasia is provided by Yu et al. [47]. This group assessed these functions in 63 poststroke patients with aphasia using the second edition of the Loewenstein Occupational Therapy Cognitive Assessment (LOTCA) battery and the Western Aphasia Battery (WAB). Data obtained by multiple regression analyses showed a close relationship between linguistic and multiple cognitive functions [47].

As it has been stated, there are important “nonlinguistic factors that participate in reshaping the neural networks supporting recovery of language functions in aphasia” in the concept of neural multifunctionality [32]. Medications for auxiliary use in the treatment of aphasia have shown mixed and rather moderate success, with most notable language improvement found with memantine, vasopressin, and piracetam and only when combined with behavioral speech treatment [48]. Hillis et al. [49] recently showed that stroke aphasic patients with damage in the left posterior superior temporal gyrus (pSTG) and/or superior longitudinal fasciculus/arcuate fasciculus (SLF/AF) and showed better naming outcome when administered Selective Serotonin Reuptake Inhibitors (SSRIs) for 3 months after their stroke. This suggests that the outcome might be modulated by SSRIs. There are two more reports on the possible positive effect of SSRIs on stroke patients, one showing better outcome in cognitive functions with escitalopram [50] and another in motor functions with fluoxetine; the result in both studies was independent of depression [51]. Whether this could implicate that SSRIs may modulate brain plasticity after stroke is an ongoing and exciting question. Approaching poststroke aphasia in terms of disrupted networks coupled with understanding how mechanisms of ischemic brain injury may be influenced by therapeutic interventions can offer better treatment strategies of poststroke aphasia in the future [52].

We now know that recovery from aphasia relies upon supportive, ancillary networks, serving as a reserve capacity. The brain must therefore be functionally reorganized, and when brain regions involved in recovery remain intact, the outcome is more favorable and vice versa. Neuroplasticity is the driving force for this functional reorganization, and neurorehabilitation is the science of enhancing it. Saur et al. studied patients with aphasia after stroke, by utilizing repeated functional MRI (fMRI) with parallel language testing [53]. They wanted to unravel the dynamics of reorganization in the language system from the acute to the chronic stage. Their findings indicated that brain reorganization during language recovery proceeds in three phases. Firstly, in the acute phase, there is a strongly reduced activation of the remaining left language areas. Secondly, an upregulation takes place with recruitment of homologue language zones, associated with improvement, and thirdly, there is a normalization of activation. A possible explanation of this normalization of activation reflects a consolidation in the language system.

An important question arising from studies like the previous one is whether the pattern of network reorganization for residual language function and recovery is consistent across aphasic patients. Turkeltaub et al. addressed this question performing a meta-analysis of functional neuroimaging studies of chronic aphasia after stroke, and they answered it positively [54]. Three distinct areas were recruited during recovery: spared left hemispheric areas belonging to a language network, new left hemispheric areas, and homotopic (to the left language areas) right hemispheric ones. The recruitment of new areas can either act compensatory or may hinder recovery. The authors conclude that the consistency in network recruitment can help us to form better rehabilitation protocols by targeting these networks. What we have learned so far from the neurorehabilitation of language functions is that aphasic patients need a holistic cognitive neurorehabilitation approach, designed and conducted by a multidisciplinary team, with neuroimaging and neuromodulation playing a protagonistic role [55].

## 9. Language and Neuromodulation

Transcranial magnetic stimulation (TMS) is a neurostimulatory and neuromodulatory technique, based on the principle of electromagnetic induction of an electric field in the brain. It has behavioral consequences and therapeutic potentials, capable of evoking long-lasting cumulative plastic changes in the stimulated cortex and also in the remote functionally interconnected areas, enhancing the brain's functional capacity and showing beneficial effects on cognitive performance in healthy persons and in patients with various neurological diseases [56]. For aphasia recovery, rTMS (repetitive TMS) has been used to inhibit activation of functional networks contralateral to the lesion (in the unaffected hemisphere, transcallosal inhibition) that actually hinders recovery. This effect can be visualized by neuroimaging [57]. Targeting these areas (and more specifically the triangular part of the right inferior frontal gyrus (IFG)) with low-frequency, inhibitory rTMS has a positive effect on language recovery in patients with aphasia following stroke [58, 59]. An important point for rTMS application is the need to stimulate the same cortical area, and this can be achieved by using brain MRI data of the treated individual to calculate the correct positioning of the coil with new generation rTMS devices by utilizing neuronavigation techniques.

Another promising neurostimulation technique is transcranial direct current stimulation (tDCS), which has already been used for improving language functions during the last decade [60]. Martin et al. investigated the behavioral and neural effects of tDCS during simultaneous fMRI in healthy young and older participants, performing semantic fluency and motor speech tasks [61]. During the stimulation conditions, performance in these tasks significantly and comparably improved in both age groups. They identified tDCS effects in the ventral and dorsal anterior cingulate regions, with an additional effect of enhanced left network laterality on older adults. Fiori et al. investigated how tDCS modulates connectivity in the language networks of healthy individuals [62]. They combined a verb learning paradigm during functional neuroimaging with simultaneous anodal tDCS over the left inferior frontal gyrus (IFG) in healthy human volunteers. They found that stimulation with anodal tDCS significantly resulted in improved performance and also caused alteration (decrease) of task-related functional connectivity between the left IFG and the right insula. This demonstrated that the behavioral improvements induced by anodal tDCS were related to an altered connectivity within a large language network. It might appear paradox, of how implementation of anodal tDCS, which has known excitatory effects, results in improved performance and also in decreased activity over the targeted left IFG. Nevertheless, this phenomenon has also been described in other studies. Holland et al. [63], in one of the few studies in the literature combining tDCS with neuroimaging techniques, investigated in healthy subjects the effect of anodal tDCS over the left IFC during concurrent fMRI in picture naming. They found that, relative to sham, it significantly facilitated the response, while on the neural level, it significantly decreased task-related activity in the stimulated inferior frontal regions. In another study, Meinzer et al. [64] targeted the left IFC in healthy elderly adults using fMRI and concurrent anodal tDCS, to assess immediate effects on cognition, with a group of younger adults serving as controls. The stimulation significantly improved performance in older adults and significantly reduced task-related hyperactivity in bilateral prefrontal cortices, inducing a more “youth-like” connectivity pattern during RS-fMRI. The authors suggest that tDCS not only modulated endogenous low-frequency oscillations in the targeted brain area but also spread to functionally connected areas of the language network. The observed reduced activity over the left frontal regions in all the above-mentioned studies can be explained as a tDCS-induced neuronal adaptation over these areas resulting in behavioral improvement. This adaptation is probably a regulation of overactivity of the targeted areas, representing a “priming” effect in neuronal level [63, 65].

Evidence is now emerging that significant improvement can be achieved in patients with poststroke aphasia combining tDCS neuromodulation and behavioral interventions [66–68]. Compared to rTMS, tDCS has certain practical advantages. tDCS devices are portable and affordable and could be easily applicable even on a home basis, making them widely available for neurorehabilitation and telerehabilitation interventions. tDCS can be applied on either the left (anodal-excitatory) or the right (cathodal-inhibitory) hemispheres, but it can also be prompted for bilateral concurrent stimulation [69]. Of course, its effectiveness and safety for various conditions must first be established, and permissions from regulatory authorities must be obtained. Furthermore, a consensus on where favorable brain changes occur to support aphasia recovery does not yet exist [68].

## 10. Conclusions

In the past, language representation in the brain was considered as being modular, and under this concept, speech treatment was limited to linguistic tasks and conducted separately for each module (i.e., naming or syntax). We now know that language functions stream widely throughout our brain and are interconnected with many other brain functions. We also recognize the brain's multifunctionality and the mechanisms for its functional reorganization. This new knowledge, coupled with technological progress, with new sophisticated and widely available and affordable tools for neuroimaging and neuromodulation, as well as telerehabilitation, will shift clinical neuroscience to an era of improved therapeutic strategies for people living with language and communication disorders.

## Figures and Tables

**Table 1:** Dual stream model: main hubs and white matter connections of the dorsal and ventral pathways for language processing.

	Dorsal stream (left dominant)	Ventral stream (bilaterally distributed)
Main “hubs”	(i) Inferior frontal gyrus (ii) Ventral portions of the precentral gyrus (iii) Anterior portions of the insula (iv) Posterior sector of the insula (v) Ventral portions of the supramarginal gyrus (vi) Area Spt	(i) Superior temporal gyrus (STG) (ii) Superior temporal sulcus (STS) (iii) Middle and inferior temporal gyri (MTG/ITG)—anterior temporal lobe (ATL)

Main fascicles	(i) Articulate fasciculus (AF) (ii) Posterior components of the superior longitudinal fascicle (SLF)	(i) External capsule (EC) (ii) Inferior fronto-occipital fascicle (IFOF) (iii) Inferior longitudinal fascicle (ILF) (iv) Uncinate fascicle (UF)
